# Detection, Speech Recognition, Loudness, and Preference Outcomes With a Direct Drive Hearing Aid: Effects of Bandwidth

**DOI:** 10.1177/2331216521999139

**Published:** 2021-04-19

**Authors:** Paula Folkeard, Maaike Van Eeckhoutte, Suzanne Levy, Drew Dundas, Parvaneh Abbasalipour, Danielle Glista, Sumit Agrawal, Susan Scollie

**Affiliations:** 1 National Centre for Audiology, Western University, London, Ontario, Canada; 2 Technical University of Denmark, Lyngby, Denmark; 3 Rigshospitalet, Copenhagen University Hospital, Denmark; 4 Earlens Corporation, Menlo Park, California, United States; 5 School of Communication Sciences & Disorders, Western University, London, Ontario, Canada; 6 Department of Otolaryngology-Head and Neck Surgery, Western University, London, Ontario, Canada; 7 Department of Medical Biophysics, Western University, London, Ontario, Canada; 8 Department of Electrical and Computer Engineering, Western University, London, Ontario, Canada

**Keywords:** extended bandwidth, direct drive, hearing aids, loudness perception, speech perception

## Abstract

Direct drive hearing devices, which deliver a signal directly to the middle ear by vibrating the tympanic membrane via a lens placed in contact with the umbo, are designed to provide an extension of audible bandwidth, but there are few studies of the effects of these devices on preference, speech intelligibility, and loudness. The current study is the first to compare aided speech understanding between narrow and extended bandwidth conditions for listeners with hearing loss while fitted with a direct drive hearing aid system. The study also explored the effect of bandwidth on loudness perception and investigated subjective preference for bandwidth. Fifteen adult hearing aid users with symmetrical sensorineural hearing loss participated in a prospective, within-subjects, randomized single-blind repeated-measures study. Participants wore the direct drive hearing aids for 4 to 15 weeks (average 6 weeks) prior to outcome measurement. Outcome measures were completed in various bandwidth conditions achieved by reducing the gain of the device above 5000 Hz or by filtering the stimuli. Aided detection thresholds provided evidence of amplification to 10000 Hz. A significant improvement was found in high-frequency consonant detection and recognition, as well as for speech in noise performance in the full versus narrow bandwidth conditions. Subjective loudness ratings increased with provision of the full bandwidth available; however, real-world trials showed most participants were able to wear the full bandwidth hearing aids with only small adjustments to the prescription method. The majority of participants had either no preference or a preference for the full bandwidth setting.

Direct drive hearing aid technology has been developed that sends an amplified, processed signal into the umbo of the tympanic membrane. This multicomponent system by Earlens (Earlens Corporation, Menlo Park, CA) is comprised of a lens, a sound processor, and an emitter. The emitter, which is housed in a vented ear-tip, couples to the sound processor which rests behind the ear and sends the signal from the processor to the lens. Earlier iterations of the device used light as the means to send the signal; however, since April 2019, commercial products have incorporated electromagnetic energy which the company reports as providing more stability of sound transference, with the transmission from the processor to the lens being affected less by jaw and facial movement (Dundas & Levy, 2020). The lens itself includes a perimeter platform, umbo platform, detector, and microactuator (Gantz et al., 2017) and is placed in the ear canal such that the platform makes direct contact with the umbo of the malleus. This device has been designed to deliver a signal directly to the middle ear system, with the goal of transmitting a broad bandwidth of audible sound from 125 to 10000 Hz for mild-to-severe sensorineural hearing losses while maintaining a vented fitting (Puria et al., 2016). The direct drive aid can be adjusted for each user’s hearing and mechanical coupling using a modified version of the Cambridge Method for Loudness Equalization 2—high-frequency (CAM2) prescription method (Arbogast et al., 2019; Moore et al., 2010) which provides targets for frequencies up to 10000 Hz. In the present article, we consider the usefulness of this extra bandwidth.

## Bandwidth and Suprathreshold Hearing Aid Outcome

Previous studies investigating the influence of bandwidth on suprathreshold measures of speech understanding and discrimination in quiet and noise in adults with sensorineural hearing loss have shown that providing extended high-frequency amplification to adults with hearing loss can improve speech understanding in both quiet and noise (Baer et al., 2002; Füllgrabe et al., 2010; Hornsby et al., 2011; Hornsby & Ricketts, 2003; Levy et al., 2015; Seeto & Searchfield, 2018; Turner & Henry, 2002; Van Eeckhoutte et al., 2020; Vickers et al., 2001). However, several studies found that an increasing severity of hearing loss or the presence of cochlear dead regions can limit the benefit provided by the extended high frequencies (Amos & Humes, 2007; Ching et al., 1998; Hogan & Turner, 1998; Hornsby et al., 2011; Vickers et al., 2001).

Studies have also shown that degree of hearing loss can influence sound quality and preference for extended bandwidth. Most studies have shown either subjective preference and higher sound quality, or no significant aversion to the provision of extended high frequencies in listeners with normal hearing and less severe hearing losses (Arbogast et al., 2019; Brennan et al., 2014; Füllgrabe et al., 2010; Moore, 2012; Moore et al., 2011; Moore & Tan, 2003; Ricketts et al., 2008; Seeto & Searchfield, 2018; Van Eeckhoutte et al., 2020). However, there are some studies that suggest there can be a negative impact of bandwidth on sound quality and preference based on stimuli used or slope of hearing loss. Ricketts et al. (2008) found listeners with hearing loss with steeply sloping high-frequency hearing losses tended to prefer a more restricted bandwidth (5.5 kHz vs. 9 kHz). Using music stimuli, Moore (2012) found that, for participants with hearing loss, there was variability in bandwidth cutoff preference between 5, 7.5, and 10 kHz and that preference for the higher cutoff corresponded to a shallow sloping high-frequency audiogram. Brennan et al. (2014) found extended bandwidth to 11 kHz was preferred over both restricted bandwidth (5 kHz) and nonlinear frequency compression for adult participants regardless of degree of hearing loss for speech stimuli; however, participants with more hearing loss were less likely to prefer the extended bandwidth for music stimuli.

Loudness perception may also vary with audible bandwidth. A recent study of loudness and bandwidth with acoustic hearing aids fitted to the Desired Sensation Level v5.0 adult targets (Scollie et al., 2005) found that improved high-frequency audibility can increase loudness perception particularly for high-level sounds (Van Eeckhoutte et al., 2020). This is in agreement with others who have studied the contributions of specific frequency bands on the perception of loudness (Jesteadt et al., 2017; Thrailkill et al., 2019) and who concluded that higher-frequency components of broadband sounds, when audible, dominate loudness perception in adults with sensorineural hearing loss.

## Bandwidth and Hearing Aid Output

Previous evaluations of conventional air conduction hearing aids have revealed upper bandwidth limits of 5000–6000 Hz in the early 2000s (Moore et al., 2001) with more recent measures revealing the capability of up to 7000–8000 Hz (Van Eeckhoutte et al., 2020) and 10000 Hz (Seeto & Searchfield, 2018). Variability in the achieved bandwidth in a fitted device may depend on limitations of the ear, degree of hearing loss, the fitted settings, target prescription and fitting methods, measurement methods, or a combination of these factors so that a hearing aid may have a nominal bandwidth that includes, for example, 100–10000 Hz but not all of this energy is audible to the listener. Seeto and Searchfield (2018) suggest that the variability in outcomes with extended bandwidths may be attributable to the lack of a clear definition of the output frequency limits of hearing aid fittings. One method used to quantify the fitted audibility provided to an acoustic hearing aid user for a speech signal using probe-tube microphone measures is the Maximum Audible Output Frequency (MAOF) range (Alexander, 2015; Kimlinger et al., 2015; McCreery et al., 2014). The MAOF range has been used in several studies to quantify the upper limits of audible bandwidth with commercially available hearing aids (Scollie et al., 2016; Van Eeckhoutte et al., 2020) and was used in this study to determine the audible bandwidth of the participants’ previously worn air conduction hearing aids.

Because the direct drive hearing device does not produce acoustic output, electroacoustic measures used to determine the bandwidth of a conventional hearing aid are not feasible. Puria et al. (2016) used temporal-bone measures to determine the maximum equivalent pressure output and stable gain of a direct drive system. Their results indicated that direct drive is expected to provide a passband to the temporal bone from 100 to 10000 Hz. The intended bandwidth of direct drive hearing aids, if successfully provided to patients, may provide more audibility than has been typically available from air conduction hearing aids in the past, particularly for fittings that incorporate significant venting. The Earlens device drives the tympanic membrane lens via an emitter housed in a vented ear-tip. No acoustic energy is delivered from this system, so the typical feedback loop created by leakage from acoustic output of an air conduction hearing aid receiver through the vent to the hearing aid microphone does not occur. Feedback generated as a result of mechanically stimulating the umbo can occur but tends to be significantly lower than that from vented acoustic hearing aids and, as a result, the direct drive system may result in less feedback than an acoustic sound source in the ear canal (Khaleghi & Puria, 2017).

This system also allows low-frequency output to be transmitted to the umbo without occluding the ear. Struck and Prusick (2017) compared the effective bandwidth and maximum gain before feedback of the Earlens versus six acoustic receiver-in-the-canal hearing aids fitted to default settings in simulated conditions and found that a broader bandwidth was achieved in both the low and high frequencies for the direct drive device. Provision of this extended bandwidth in direct drive fittings has been confirmed by previous studies using measures of aided in situ thresholds up to 10000 Hz (Arbogast et al., 2019; Fay et al., 2013; Gantz et al., 2017). Measures of threshold-level responses to tonal test signals, however, can only confirm audibility of low-level signals and do not provide a direct evaluation of the suprathreshold sound from the direct drive system. Evaluation beyond that of aided functional gain is desirable to consider aided outcomes such as loudness, speech recognition, and/or sound quality of real-world signals such as speech and music (Humes, 2003; Stelmachowicz et al., 2002). Levy et al. (2015) suggested that more research was needed to determine the benefits of extended bandwidth above the range typically seen with acoustic hearing aids. Gantz et al. (2017) reported aided versus unaided benefit using the direct drive hearing devices on measures of word recognition in quiet and sentence recognition in noise; however, the effects of the potentially increased bandwidth from fitted direct drive devices on speech recognition, loudness, and preference have not been evaluated in previous studies. Benefit in contrasting bandwidth conditions is considered indirect measures of the available frequency response, as an index of achievable gain and performance on an individual level (Levy et al., 2015; Seeto & Searchfield, 2018). Measures that relate to functional communication are important for understanding how performance with a direct drive system varies with aided bandwidth, and studies in users of clinically available devices are necessary to determine whether the speculative benefits of bandwidth from lab- and headphone-derived studies can be achieved in wearable devices.

The purposes of this study were (a) to assess the functional bandwidth of the Earlens direct drive hearing aid by measuring aided sound field thresholds; (b) evaluate suprathreshold aided speech and loudness perception obtained with the Earlens device across bandwidth conditions that compare to past studies with acoustic hearing aids in listeners who had received a trial with the Earlens system; and (c) determine listener preference for restricted versus narrow bandwidth when wearing the Earlens system. In these tasks, we compared outcomes with the full bandwidth condition compared with narrow bandwidth test conditions. This method is consistent with previous studies of bandwidth (Brennan et al., 2014; Füllgrabe et al., 2010; Stelmachowicz et al., 2007; Van Eeckhoutte et al., 2020). In a companion study (Vaisberg et al., 2021), we investigated the Earlens device using sound quality ratings and found that the wider bandwidth that included both low- and high-frequency energy received higher sound quality ratings. The current study extends these results to examine measures of detection, benefit, loudness, and preference.

## Methods

This study was approved by the Western University Human Research Ethics Board (109433) and Lawson Health Research Institute (R-18-057).

### Participants

Potential participants were screened for study inclusion and exclusion criteria (Fay et al., 2013) prior to enrollment (Table 1). Audiometric inclusion criteria followed Arbogast et al. (2019) (i.e., mild-to-severe sensorineural hearing loss with suprathreshold word recognition on the NU-6 word list ≥60% and normal tympanometry). This study required these criteria bilaterally.

**Table 1.** Inclusion and Exclusion Criteria for Participant Candidacy.

Inclusion:
The participant must:• Be an adult ≥18 years. • Be a fluent speaker of English. • Have mild-to-severe sensorineural hearing loss to 8000 Hz with word recognition scores ≥60%.• Have symmetrical hearing (<15 dB HL difference between thresholds in the left and right ears with the allowable exception of one frequency) • Have a normal tympanogram.
Exclusion:
The participant must not have any known or active medical issues that would preclude having a hearing device, including: • An abnormal tympanic membrane (deemed perforated, inflamed, or has dimeric or monomeric area, or in any other way abnormal). • An abnormal middle ear or a history of prior middle ear surgery other than tympanostomy tubes. • An ear canal anatomy that prevents the physician from seeing an adequate amount of the tympanic membrane. • An anatomical configuration of the external auditory canal that prevents satisfactory placement of the lens. • A history of chronic and recurrent ear infections in the past 24 months. • A rapidly progressive or fluctuating hearing impairment. • Diagnosed with having a compromised immune system which may impact the tissue of the auricle or ear canal, such as keratosis obturans, ichthyosis, eczema of the auricle or ear canal, or have ever received radiation of the head or chemotherapy for cancer within the past 6 years.

Twenty-eight participants from London, Ontario and the surrounding area signed letters of informed consent. Thirteen of the 28 participants enrolled but did not complete the trial for the following reasons: small exostosis that prevented inclusion and was not apparent until after ear cleaning (*n* = 1); visualization of a weak spot on the tympanic membrane at the time of ear cleaning (*n* = 1); voluntary withdrawal of permission prior to impression (*n* = 1); participant could not tolerate the impression process (*n* = 1); initial impression was not successful and participant declined to return (*n* = 2); impressions were taken successfully, but a device could not be fabricated due to size and shape restrictions (*n* = 6); and device was fitted but the participant withdrew due to autophony when singing (*n* = 1). The remaining 15 participants completed the entire protocol. Of these, seven participants were female (mean age: 72 years, range: 66–78); eight were male (mean age: 72.4 years, range: 68–86).

### Otologic Assessments

**Figure 1.**
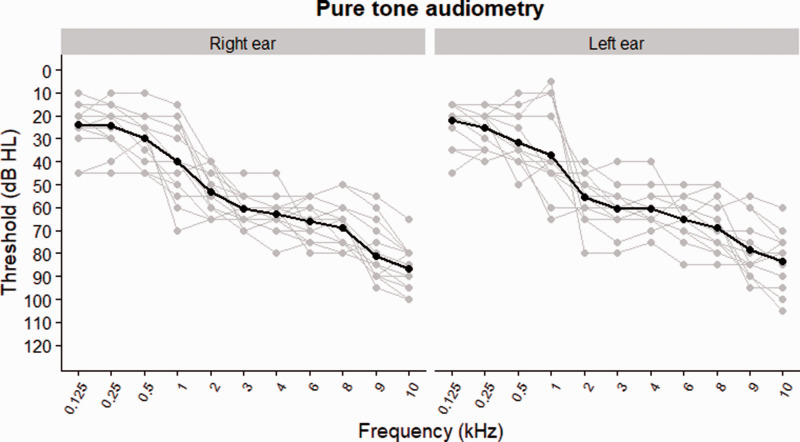
Pure Tone Detection Thresholds for the Left and Right Ear by Air Conduction (in Gray), Along With the Mean Thresholds (in Black) for All Participants (*N* = 15).

All participants had audiometric testing for the octave and interoctave frequencies spanning 125 through 20000 Hz. Test frequencies for 125 through 8000 Hz were measured with insert earphones connected to foam tips, and the extended high frequencies were tested with Sennheiser HDA300 circumaural headphones. Testing was completed in a double-walled sound booth, using a GSI-61 audiometer that had been calibrated to meet ANSI S3.6 (2010) standards. Participants’ audiometric thresholds are shown in Figure 1. Evaluation of the middle ear system was completed using the Titan wide band reflectance system.

### Amplification History

Participants’ amplification experience with air conduction hearing aids ranged from 3 months to 23 years (*M* = 9.8 years, *SD* = 7.4 years). Probe-tube microphone measures of participants’ own aids were completed to measure the output of the hearing aids at 55, 65, and 75 dB sound pressure level (SPL) using the International Speech Test Signal (Holube et al., 2010). From the aided International Speech Test Signal spectrum, the MAOF of the participants’ own aids was determined. An example of a participant’s calculated MAOF for the 65 dB SPL speech signal is presented as supplemental digital content (S1). The mean MAOF for aided conversational speech at 65 dB SPL was 4185 and 5719 Hz for the RMS or peak levels of speech, respectively (data for other levels are presented as supplemental digital content S2). Results indicated that while wearing their own hearing devices, on average, participants received audible midlevel speech to just below 6000 Hz, ranging from about 2500 to 10000 Hz across participants and that speech audibility varied with speech level.

### Ear Impression Procedure

**Figure 2.**
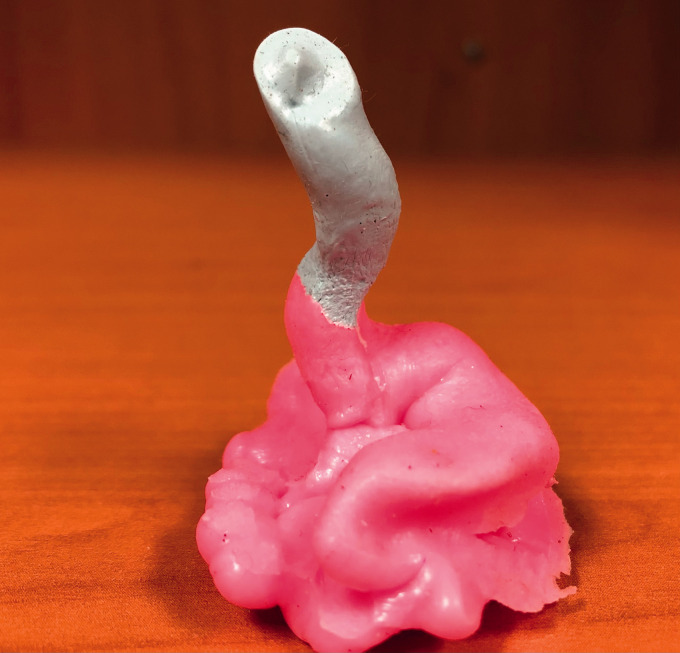
Two-Stage Deep Canal Impression. The blue material is applied directly on the tympanic membrane, and the imprint of the umbo can be clearly seen on the resulting impression.

All participants completed a deep ear canal impression procedure bilaterally, as required for the custom manufacturing of the direct drive lens and the ear-tips. Ear cleanings, followed by impressions, were conducted by an experienced neurotologist, following recommended procedures. Two-step deep ear canal impressions provided casts of the external ear canal including the tympanic membrane (Figure 2). Otoscopic examination was performed following removal of the impression to monitor the status of the ear. There were no serious adverse side effects from the impressions during the study. Mild side effects included small bilateral ear canal hematomas that self-resolved with one participant and temporary post-impression autophony with another participant. Note that this was not the same participant who voluntarily withdrew from the study postfitting due to autophony while singing. Examination of the impression was performed to ensure that a complete representation of the outer ear had been obtained.

### Device Fitting and Programming

All participants were fitted with Earlens direct drive hearing devices between July 2018 and July 2019. Devices used light-based signal transmission. On the day of fitting, the lens was placed on the tympanic membrane by the same neurotologist who had performed the ear impression. Medical-grade mineral oil was applied to the ear canal to facilitate device retention and prevent debris buildup in the ear canal. Following placement, the external processor was fitted and programmed by an audiologist using proprietary Earlens Fitting software (version 1.7.2). The fitting software uses the audiometric thresholds to compute modified CAM2 prescriptive targets. In situ detection thresholds for the frequencies 125–10000 Hz are also measured using this software to determine the signal levels transmitted from the device to the listener. The test platform allows the clinician to present pure tones (via the device) and to manually search for and bracket thresholds using 5 dB steps in a one-up, two-down Hughson-Westlake adaptive tracking procedure (Carhart & Jerger, 1959). The software uses this in situ calibration to scale the output of the system and to determine the available maximum output on a frequency-by-frequency basis. This allows for display of the audibility of signals generated by the system relative to the available dynamic range achieved on the ear. The devices were initially fitted to these targets by the software while the participant was seated in a quiet room and fine-tuned to the participant’s preference if necessary. Participants were provided with additional hearing aid programs based on the frequency response of the tuned-to-preference fitting but which varied in microphone directionality and noise reduction strength as required for use in their daily lives (e.g., music program). Participants also had access to a volume control for use in the real-world trial.

Because the application of oil during lens placement is known to cause a damping effect on hearing that is expected to subside after a few days, device programming was monitored and readjusted as follows. Each participant was seen 1–2 weeks postfitting to confirm device function and use and to address any questions. In situ calibration thresholds were reassessed at this and each subsequent appointment to ensure the stability of the fitting and to reprogram the device and/or provide modified physical fits to ensure comfortable and stable fittings if needed. On average, the final fittings fell within 2.3 dB root mean square error (RMSE) from the prescribed targets across all frequencies (125–10000 Hz) following fine tuning for preference (Table 2). Participants’ individual RMSE results ranged from (0 to 8.5 dB) with 13/15 of the fittings having an RMSE less than 6.3 dB. These results indicate that most devices were fitted to a close approximation of the target and that most users’ requests for fine tuning to preference tended to be consistent with those in Arbogast et al. (2019).

**Table 2.** Descriptive Statistics of the Average Deviation From the Moderate Target (65 dB SPL), Including Signed Mean Difference, Standard Deviation of the Signed Difference, and Calculated Unsigned RMSE Values of Four Different Frequency Ranges.

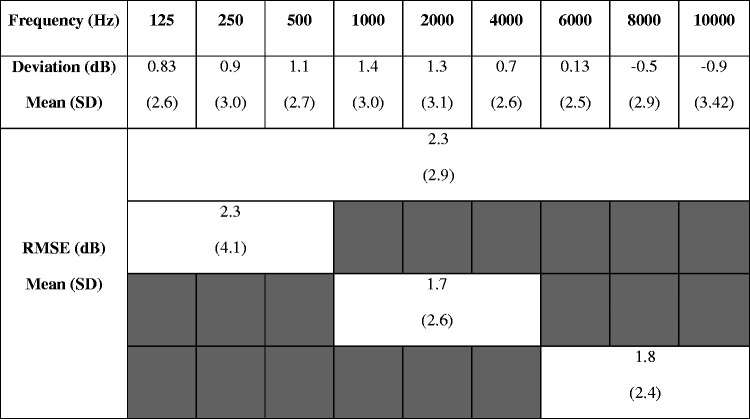

*Note*. RMSE = root mean square error.

### Real-World Trials

Following the fitting and follow-up procedures mentioned previously, participants were requested to wear the direct drive aids for a minimum of 4 weeks. The mean trial time before outcome measurement was 6 weeks (*SD *= 3.3). The majority of participants (73%) were within the range of 5–7 weeks. During this time, participants could revisit the laboratory for fine tuning or troubleshooting. Four participants returned and required remakes to the custom lens and ear-tip, which extended their wear time to 10–15 weeks before outcome measurement. One participant with a large amount of ear canal change with jaw movement required two sets of lenses and three sets of ear-tips. One participant had a new lens and ear-tip made due to a lens malfunction at the time of the fitting. Two participants had complaints of “static” that were not resolved with reprogramming and were refit with a new lens and ear-tip monaurally (*n* = 1) and new ear-tips bilaterally (*n* = 1). Troubleshooting components resulted in these four participants being seen for 2–7 additional in-lab appointments. One additional participant returned to have a noise program added for real-world use. In situ light calibration on the day of outcomes measurement confirmed stable fittings for all participants including those who had had the lens and ear-tip remakes.

### Posttrial Outcome Measures

Following the trial period, outcome measures were administered to assess the effects of extended bandwidth on aided outcomes. The battery included aided tone detection thresholds, speech recognition, aided loudness perception, and preference. The hearing aids were set to an omnidirectional mode with advanced signal processing disabled. Two programs for this test battery were created: (a) full bandwidth, with the participants’ real-world trial frequency response and (b) narrow bandwidth with a frequency response programmed to provide no output above 5000 Hz. All outcome measures except loudness perception were completed in both hearing aid programs. The 5000 Hz cutoff for the narrow bandwidth condition was chosen to be comparable to narrowband conditions reported for acoustic hearing aid fittings (Füllgrabe et al., 2010; Kimlinger et al., 2015; McCreery et al., 2014). The test conditions (full bandwidth versus narrow bandwidth) were not revealed to the participant during outcome measures.

For the loudness rating task, listeners wore the hearing devices in the full bandwidth program, and the stimuli were filtered to create four different bandwidths: (a) 123–10869 Hz; (b) 123–4455 Hz; (c) 313–10869 Hz; and (d) 313–4455 Hz. These filter conditions were selected to match those used by Moore and Tan (2003). For this measure, we filtered the stimuli with these filter conditions rather than using the hearing aid programming in an attempt to match Moore and Tan’s experimental conditions. Sound quality ratings with these filter conditions have been previously reported in a companion study (Vaisberg et al., 2021).

The order of tests and conditions was randomized across listeners and test sessions. Testing was completed in one or two test sessions depending on scheduling and to prevent fatigue. If testing was completed on multiple days, repeated tests were scheduled on the same day to avoid differences in rest periods between conditions. All aided testing was completed in a standard 283 cm × 305 cm double-walled sound booth with a reverberation time (RT60) of 0.1 s. The participant was seated with access to a computer monitor and mouse. Seven loudspeakers surrounded the listener at horizontal angles of 45°, 90°, 135°, 180°, 225°, 270°, and 315° relative to the look direction at a distance of 110 cm and a height of 120 cm (floor to speaker centre). An eighth speaker at 0° was located at a distance of 115 cm and a height of 125 cm to accommodate conditions that required both speech and noise from 0°. In those conditions, the 315° speaker was moved to be directly in front of the 0° speaker. Listeners wore the Earlens system in both ears while the test battery was completed.

#### Detection Thresholds

Detection thresholds were conducted in a calibrated sound field using frequency-modulated tones from 125 to 10000 Hz produced by a clinical audiometer (GSI-61). All detection thresholds were bracketed using 5 dB steps and one-up, two-down adaptive tracking using the Hughson-Westlake procedure. Detection thresholds were measured unaided (i.e., pre-lens fitting with no devices in the ears) and aided (i.e., with lenses in place and hearing aids fitted and turned on). Aided thresholds were measured twice, once with the hearing aids set to the full bandwidth condition and also with the hearing aids set to the narrow bandwidth condition.

#### Speech Recognition

Speech recognition was assessed using three measures; one using sentences, one using nonsense syllables, and one measuring word-final /s/ detection, described later. Each test was completed twice, with the participant wearing the hearing devices in each of the full and narrow bandwidth programs.

Sentence recognition in noise was measured with the Hearing in Speech Test (HIST). This test uses sentences and bracketing procedures from the Hearing In Noise Test (Nilsson et al., 1994), with rerecorded stimuli that provide bandwidth to 20000 Hz and two-talker babble rather than steady-state background noise (Levy et al., 2015). Bracketing of the stimulus level was administered with software to measure the Reception Threshold for Sentences at a 50% correct level. Masker level was held constant at 65 dB(A). Three loudspeaker configurations were used: (a) target speech and two masking talkers from zero degrees azimuth; (b) target speech from zero degrees and two masking talkers from ±45 degrees; and (c) target speech from –45 degrees and two masking talkers from +45 degrees. The third configuration used an asymmetric configuration in which the two types of stimuli were spatially separated. This condition was included because it has been shown to be sensitive to bandwidth effects in a previous investigation in which listeners were tested with simulated amplification over headphones (Levy et al., 2015).

Consonant recognition in noise was measured with the University of Western Ontario Distinctive Features Differences test (Cheesman & Jamieson, 1996). The University of Western Ontario Distinctive Features Differences test presents the 21 consonants of English for two male and two female talkers. All test stimuli begin with the vowel sound /æ/ and end with /Il/. The middle consonants change during the task, and the listener is asked to select the consonant that was heard from a computer display of 21 response choices (e.g., If the participant heard /æbIl/, they would choose the letter B on the screen.). Stimuli were presented at a conversational level of 60 dB SPL (ANSI, 2020; Pearsons et al., 1977) in a background of steady-state speech-shaped noise at a +6 dB signal-to-noise ratio (SNR) at zero degrees azimuth. This or similar tests at similar levels and SNRs have been used in evaluations of extended bandwidth in air conduction hearing aids (McCreery et al., 2014; Van Eeckhoutte et al., 2020). Listeners selected the perceived medial consonant on the computer screen, and responses were logged within the test software. The test was completed twice per condition (i.e., full bandwidth and narrow bandwidth) for a total of four tests. An error matrix was generated to investigate specific patterns of individual consonant recognition confusion between the two bandwidth conditions (Alexander & Rallapalli, 2017; Glista et al., 2012; Salorio-Corbetto et al., 2017; Van Eeckhoutte et al., 2020).

Aided fricative detection was measured with the University of Western Ontario Plurals Test (Glista & Scollie, 2012), which measures detection of word-final /s/ using a list of nouns in singular and plural forms. The test was administered at 55 dB SPL with a masker noise embedded in the test at 20 dB SNR, both from zero degrees azimuth. This test was completed twice per condition (i.e., full bandwidth and narrow bandwidth) for a total of four tests.

#### Aided Loudness Perception

The Contour Test of Loudness Perception (Cox et al., 1997) was used to measure aided loudness ratings, for sentences from the modified Connected Speech Test passages *Ocean and Water* (Saleh et al., 2020). With each sentence presentation, the listeners indicated their perceived loudness category on a computer screen. Seven loudness categories included the following: *Very soft, Soft, Comfortable but slightly soft, Comfortable, Comfortable but slightly loud, Loud, and Uncomfortably loud*. Presentation levels ranged from 52 to 80 dB SPL and increased in steps of 4 dB until either the maximum level was reached or a rating of *Uncomfortably loud* was given. At that point, the stimulus level reversed (descending run). Loudness ratings were scored from 1 to 7. Ratings from the ascending and descending run were averaged together to obtain one number for each stimulus level for a given test condition (Jenstad et al., 2007). Each condition was presented twice, for a total of eight loudness tasks. The loudness results for the two repetitions were averaged to obtain a single result for each test condition.

#### Preference

Subjective preference for either the full or narrow bandwidth condition was measured using a single-blind, unforced-choice, paired comparisons paradigm (Amlani & Schafer, 2009; Eisenberg et al., 1997; Punch et al., 2001). Listeners heard a broadband recording of the Dove Passage from the modified Connected Speech Test (Saleh et al., 2020). The passage was presented at 60 dB SPL from 0° azimuth. The tester alternated the hearing devices between the full narrow bandwidth programs using the manufacturer’s software. Participants were asked to state their preference for Program 1, Program 2, or declare no preference. Participants did not know the nature of the difference between the two programs. The procedure was repeated twice with the order of programs reversed on the second trial. Starting condition was counterbalanced across participants. For the analysis, the preference results were coded as follows. If the listener had the same preference in both trials, the response was coded as a *strong preference*. If the listener indicated a preference for a condition in one trial, and no preference in another trial, this was coded as a *weak preference. No preference* was coded if (a) the listener selected no preference on both trials or (b) the listener selected a preference for one condition in one trial and a preference for the other condition in the other trial. Correlation analyses were completed to determine any relations between degree and slope of hearing loss with bandwidth preference (Moore, 2012; Ricketts et al., 2008).

## Results

### Detection Thresholds

**Figure 3.**
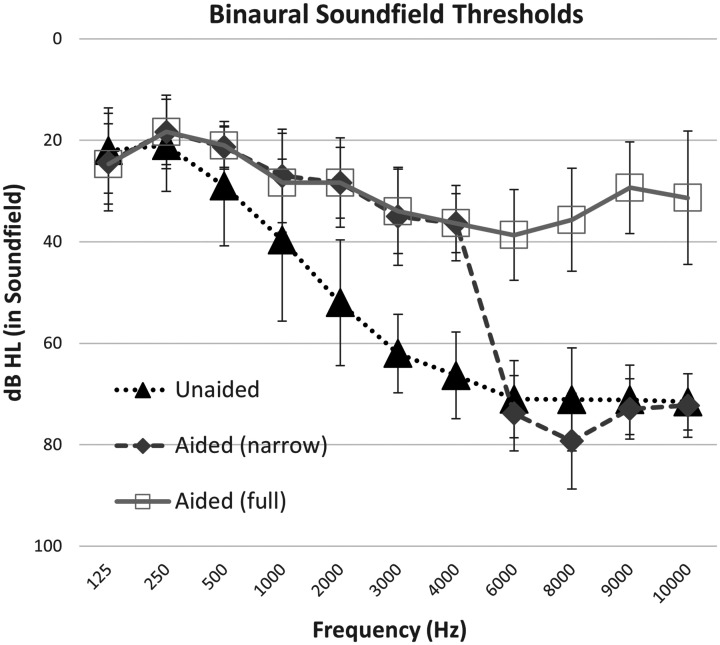
Average Binaural Detection Thresholds for Frequency-Modulated Tones in Sound Field for All Participants (*N* = 15). Error bars indicated one standard deviation from the mean. Conditions include unaided listening versus listening with the direct drive hearing aid, programmed either with settings for use in a trial period (full bandwidth) or with narrow bandwidth test condition with gain restricted above 5000 Hz.

Unaided and aided detection thresholds are shown in Figure 3. In the unaided and narrow bandwidth conditions, there were several participants with thresholds above the limits of the audiometer at the higher test frequencies: at 8000 Hz (*n* = 1), 9000 Hz (*n* = 7), and 10000 Hz (*n* = 8) in the unaided condition; and at 6000 Hz (*n* = 2) and 8000–10000 Hz (*n* = 9) in the narrow bandwidth condition. Thresholds above the limits of the audiometer were coded as the maximum presentation level for that frequency +1 dB. In the full bandwidth condition, all participants had measurable aided thresholds at all frequencies tested (125–10000 Hz).

Detection thresholds were analyzed with a repeated-measures analysis of variance, using Greenhouse–Geisser corrections to adjust for lack of sphericity. Aided thresholds were the dependent variable, and test frequency and bandwidth condition were repeated factors. There was an overall effect of test frequency, *F*(3.62, 50.64) = 86.90, *p* ≤ .001, ŋ^2^ = .85, and bandwidth condition, *F*(1.37, 19.10) = 211.31, *p* < .001, ŋ^2^ = .94, as well as an interaction between bandwidth condition and test frequency, *F*(5.19,72.71) = 76.68, *p* < .001, ŋ^2^ = .85. Frequency-specific pairwise comparisons were completed for (a) unaided versus full bandwidth to measure aided benefit and (b) narrow versus full bandwidth to test the impact of bandwidth. Aided full bandwidth thresholds were better than unaided at 500 Hz and above, through 10000 Hz (500 Hz: *p* = .023; 1000 Hz: *p* = .001; all other frequencies *p* < .001). Narrowband aided thresholds were poorer than full bandwidth aided thresholds at 6000 Hz and above (*p* < .001). At all other frequencies, narrow and full bandwidth thresholds did not differ significantly (125, 250, 500, 2000, 4000 Hz: *p* = 1.00; 1000 Hz: *p* = .311; 3000 Hz *p* = .813). These results indicate that improved detection was observed in aided versus unaided conditions and also that the narrow bandwidth programming successfully reduced high-frequency audibility, at least for the low levels tested in this task.

### Speech Recognition: Sentences in Noise

**Figure 4.**
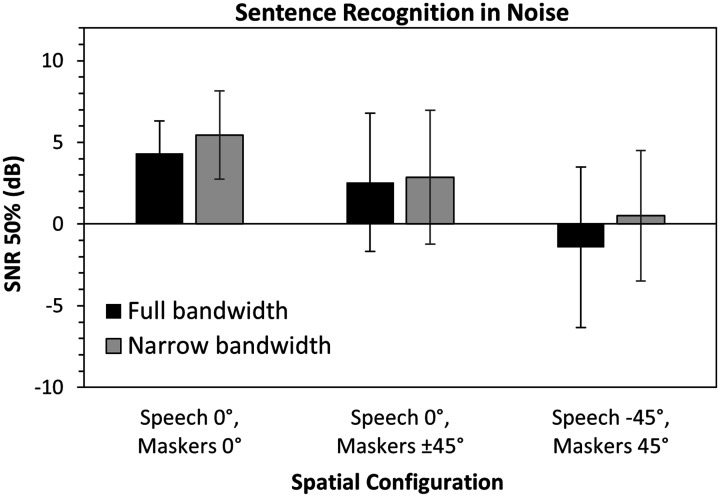
Mean Reception Threshold for Sentences in Two-Talker Babble at Three Azimuths in Full Bandwidth Versus Narrow Bandwidth (*N* = 15). Error bars indicate one standard deviation. Overall, there was a significant difference between full and narrow bandwidth conditions. SNR = signal-to-noise ratio.

Mean reception thresholds for sentences from the HIST are shown in Figure 4. An analysis of variance was completed to assess the effect of spatial configuration (i.e., azimuths) and bandwidth (full, narrow) as repeated measures. Results indicated a significant main effect of spatial configuration, *F*(1.95, 27.34) = 27.34, *p* ≤ .001, ŋ^2^ = .66, and bandwidth, *F*(1, 14.00) = 4.98, *p* = .042, ŋ^2^ = .26. The interaction between bandwidth and spatial configuration was nonsignificant, *F*(1.93, 27.01) =  3.10, *p* = .063, ŋ^2^ = .184. On average, participants were able to recognize HIST sentences with 1.1 dB more background noise in the full bandwidth condition versus the narrow bandwidth condition, averaged across spatial configurations.

### Speech Recognition: Consonants in Noise

**Figure 5.**
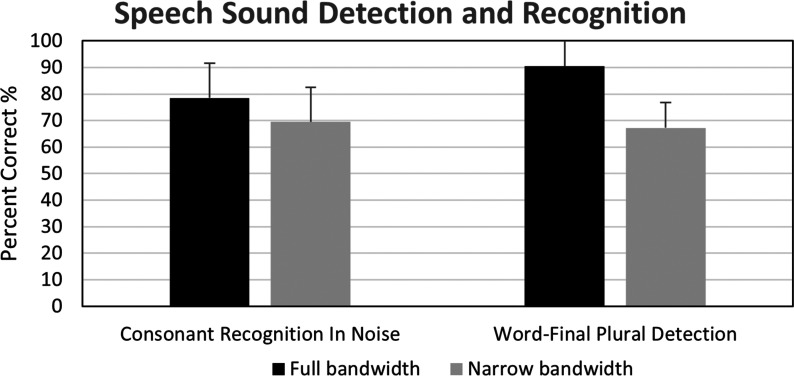
Mean Percentage Correct Scores for Consonant Recognition in Noise and Word-Final Plural Detection in the Full Versus Narrow Bandwidth Conditions (*N* = 15). Error bars indicate one standard deviation. There was a significant difference between the two bandwidth conditions for both outcome measures.

Figure 5 displays the mean percent correct scores for consonant recognition in full and narrow bandwidth conditions. For analysis, the percent correct scores were converted to rationalized arcsine units (RAU) (Studebaker, 1985). A paired samples *t* test was completed on the RAU scores. Scores were significantly better by 10.2 RAU in the full bandwidth condition (*M* = 79.6*, SD* = 15.6) compared with the narrow bandwidth condition (*M* = 69.4, *SD* = 13.8); *t*(14) =  –2.83, *p* = .01.

The differences in error patterns for individual consonants between the two bandwidth conditions were analyzed on a confusion difference matrix by subtracting the number of correct trials in the narrow bandwidth condition from those in the full bandwidth condition, per consonant (Table 3). With four talkers and two repetitions, each consonant was presented 120 times across the 15 listeners. The highest rate of differences in confusions was observed for /s/ with 65% (78/120 trials) more correct identifications in the full bandwidth condition. The most frequent confusion in the narrow bandwidth condition was perceiving /s/ as/f/(57/120 trials). Similarly, /z/, the voiced cognate of /s/, had 70/120 more correct identifications in the full bandwidth condition. The next most frequent confusions included the perception of /z/ as /v/ in 37/120 trials and /t/ as /k/ in the narrowband condition (34/120 trials). Taken together, these improvements in consonant recognition indicate a higher rate of accuracy for those phonemes with higher frequency content when using the direct drive hearing system at full bandwidth.

**Table 3.** Difference in Consonant Confusions Between the Full Bandwidth Minus the Narrow Bandwidth Conditions.

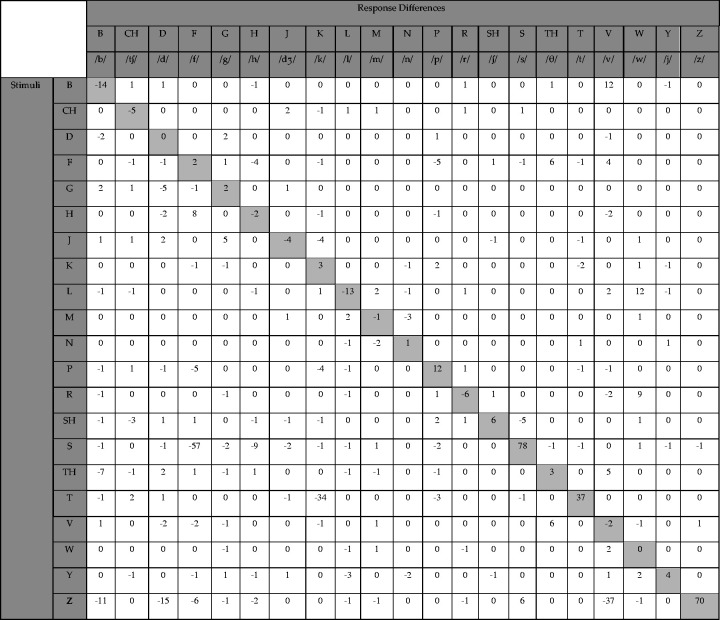

*Note*. Positive values on the diagonal indicate better performance in the full bandwidth condition. Negative values outside the diagonal indicate more confusion between consonants in the narrowband condition.

### Speech Recognition: Word-Final Plural /s/ Detection

Figure 5 also displays the mean percent correct scores on the University of Western Ontario Plurals test, for full and narrow bandwidth conditions. Performance improved by 25 percentage points in the full bandwidth compared with the narrow bandwidth condition. For analysis, percent correct scores were converted to RAU, and a paired samples *t* test comparing bandwidth conditions was completed. Results indicated a significant improvement in the full bandwidth condition (*M* = 97.3, *SD* = 16.4) compared with the narrow bandwidth condition (*M* = 65.4, *SD* = 9.5), *t*(14) =  –6.2, *p* = .<001.

### Loudness Ratings

**Figure 6.**
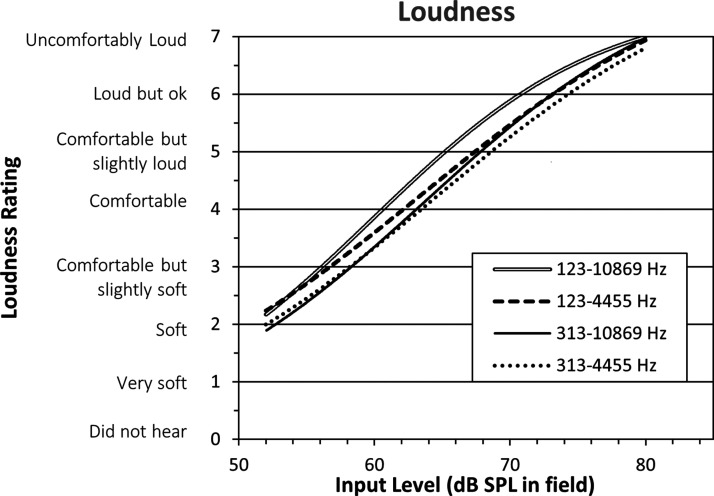
Fitted Sigmoidal Functions From Loudness Judgments in Four Different Bandwidths Ranging From Full to Narrow Conditions (*N* = 15). Condition 123–10869 Hz was significantly louder than the other three bandwidth conditions. There was also a significant difference between conditions 123–4455 Hz and 313–4455 Hz. SPL = sound pressure level.

Figure 6 displays a sigmoidal fit to the bilateral aided loudness ratings from all participants for each condition. Perceived loudness increased with increasing stimulus level for all four conditions, with the largest bandwidth condition generally having higher loudness judgments than the other conditions. A linear mixed-effects model was completed with participant as a random effect and bandwidth and level as fixed effects. Adding an interaction did not improve the model. This was tested by comparing the models with the log-likelihood. Adding level and bandwidth to the model significantly improved it, χ^2^ (1) = 1087.08, *p* < .001 and χ^2^ (3) = 27.75, *p* < .001, respectively, while adding the interaction did not significantly further improve the model, χ^2^ (3) = 4.04, *p* = .26. The reference bandwidth was 123–10869 Hz. Post hoc paired *t* tests with Bonferroni correction were chosen to further examine differences between pairs of bandwidth conditions. Results showed that the reference bandwidth condition (123–10869 Hz) was significantly louder than the other three bandwidth conditions (*p* < .001). There was also a significant difference between the two narrowest bandwidth conditions (123–4455 Hz vs. 313–4455 Hz, *p* < .001), indicating that providing the low-frequency band yielded increases in loudness judgments. There were no significant differences found between the loudness judgments of the remaining conditions (*p* > .05).

The input level at which the categories “Comfortable, but slightly soft” and “Comfortable, but slightly loud” were reached for each of the conditions was computed to summarize the functional magnitude of the differences in loudness judgment across conditions. The largest bandwidth condition (123–10869 Hz) reached these loudness categories at input levels that were 2.2 and 3.4 dB lower than in the narrowband condition.

### Preference

**Figure 7.**
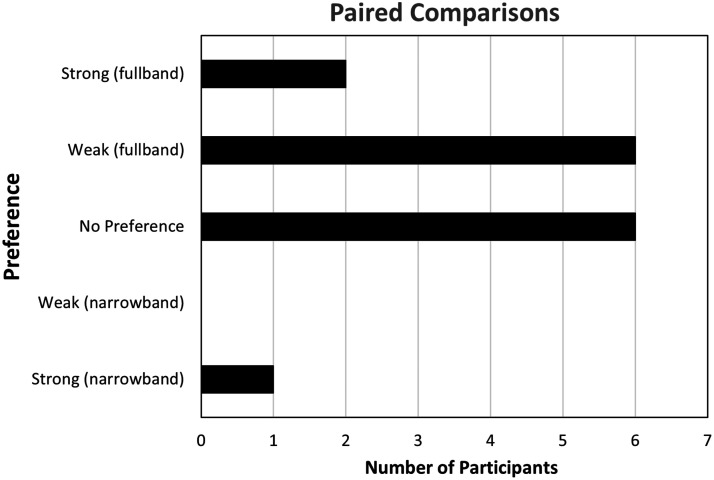
The Number of Listeners From the Group of 15 Who Indicated Whether They Preferred Full or Narrow Bandwidths, or Had No Preference, in a Paired Comparison Task.

Paired comparisons testing (Figure 7) indicated that 8 of the 15 listeners had preference for the full bandwidth condition, with 6 listeners having a weak preference and 2 having a strong preference. Of the remainder, 6 listeners had no preference, and 1 listener had a strong preference for the narrowband condition. A one-sample Shapiro–Wilk test for normality indicated a significant skew toward preference for the full bandwidth condition (*p* = .02). Further investigation was performed to determine if audiometric pure-tone average (PTA) and/or slope were factors in preference. PTA did not relate significantly to preference, whether this was defined for four frequency PTA (500, 1000, 2000, 4000 Hz) (*r* = .35, *p* = .21) or three frequency PTA (500, 1000, 2000 Hz) (*r* = .38, *p* = .16) or PTA (1000, 2000, 4000 Hz) (*r* = .30, *p* = .28). For slope, we investigated two definitions based on previous studies: (a) the difference between 8000 Hz thresholds and 4000 Hz thresholds (Moore et al., 2011; Van Eeckhoutte et al., 2020) and (b) the difference between 12500 Hz thresholds and 4000 Hz thresholds (Ricketts et al., 2008; Van Eeckhoutte et al., 2020). Neither slope estimate was a significant predictor of preference (8000–4000 Hz: *r* =  –.13, *p* = .64; 12500–4000 Hz: *r* = .14, *p* = .64).

## Discussion

In the present study, the effects of bandwidth on speech perception, loudness, and preference were evaluated by fitting bilateral Earlens direct drive hearing devices to participants with sensorineural hearing loss. Participants in this study met recommended criteria for use of the Earlens in terms of ear size and characteristics, medical history, and audiometric thresholds. The audibility of the extended bandwidth up to 10000 Hz was confirmed with aided sound field audiometric testing, which demonstrated a significant improvement in high-frequency thresholds between the unaided and aided conditions. In this study, we examined the effects of extended bandwidth. For speech recognition and preference tasks, the bandwidth of the direct drive devices was restricted to 5000 Hz, thereby creating two test conditions: (a) the full clinical bandwidth as fitted to the participants’ hearing loss using the CAM2 fitting method and (b) a narrow bandwidth fitting. The 5000 Hz cutoff for the narrow bandwidth condition was chosen to be comparable to narrowband conditions reported for acoustic hearing aid fittings (Füllgrabe et al., 2010; Kimlinger et al., 2015; McCreery et al., 2014). Although a few recent studies (Seeto & Searchfield, 2018; Van Eeckhoutte et al., 2020) have shown that modern hearing aids can provide extended high-frequency bandwidths past 7000 Hz in field trials, measurements of our participants’ acoustic hearing aids which were from a variety of manufacturers, ranged in age from <1 year to 5 years old, and were fitted outside of the research context, in fact showed an average bandwidth range between 4185 and 5719 Hz for speech levels at 65 dB SPL, RMS or peak respectively. In this study, the clinically provided bandwidth of the participants’ own acoustic aids was relatively well-approximated by the 5000 Hz narrow bandwidth condition tested with the direct drive devices.

As noted earlier, hearing tests in the sound field confirmed improved audibility in the full versus narrow bandwidth condition for threshold of hearing with use of the direct drive devices. We also examined suprathreshold outcomes with full versus narrow bandwidth fittings, including speech sound detection and recognition and speech in noise understanding. Overall performance of speech recognition for nonsense syllables in noise significantly improved in the full versus narrow bandwidth condition, with improved recognition of consonants with high-frequency content such as the phonemes /s/ and /z/. Specifically, this improvement in fricative identification is consistent with the results published in the development of the Plurals test, where high-frequency /s/ in word-final position was detected with greater accuracy in the broadband condition (Glista & Scollie, 2012). These results agree with previously reported results on similar tests with extended bandwidth amplification and/or frequency lowering signal processing (Baer et al., 2002; Füllgrabe et al., 2010; Glista & Scollie, 2012; McCreery et al., 2014; Salorio-Corbetto et al., 2017; Seeto & Searchfield, 2018; Stelmachowicz et al., 2007; Van Eeckhoutte et al., 2020). Taken together, these results indicate that the audible bandwidth of the direct drive system supports improved recognition of high-frequency speech sounds.

One of the most common challenges for those with hearing loss is understanding speech in background noise. There was an overall benefit of extended bandwidth for sentence recognition in speech noise using the HIST. These results are consistent with findings reported by Levy et al. (2015), whose participants performed better when using a full bandwidth hearing aid setting compared with a narrow bandwidth setting simulated over headphones. The improvement in SNR 50% from the collocated condition compared with the spatially separated conditions is generally in agreement with the concept of spatial release of masking and results reported for the HIST by Levy et al. (2015), and with previous studies indicating that audible bandwidth improves spatial release from masking in listeners with hearing loss (Jakien et al., 2017).

To explore the interaction between the provision of extended bandwidth and loudness perception, the effect of bandwidth on loudness perception was measured in this study using four bandwidth conditions. Consistent with results from Van Eeckhoutte et al. (2020), perceived loudness judgments were higher for the stimuli with the broadest bandwidth and exceeded that of either the low- or high-frequency conditions. When low-frequency energy was added (e.g., comparing the 123–4455 vs. 313–4455 Hz conditions), a significant increase in loudness was observed, indicating low-frequency energy contributed to the loudness percept. This increased further when stimuli were extended in both low- and high-frequency bands (i.e., 123–10869 Hz) which indicates that high-frequency energy also contributes to loudness perception. This result is generally consistent with previous work that found contribution of energy at both low and high frequencies depending upon sensation level (Jesteadt et al., 2017; Thrailkill et al., 2019). We note that when the low-frequency energy was not present (in the conditions 313–4455 and 313–10869), loudness ratings were not significantly different. It would appear that the impact of high-frequency energy was reduced when the low-frequency band was filtered out of the signal. Differences among other conditions, although statistically significant, were within the observed range of clinically typical deviations from target (Dao et al., 2021) and were within one audiometric step size. Compared with these reference points, this difference (2.2–3.4 dB in input level) is unlikely to be considered clinically significant and indicates that while the extended bandwidth signal was perceived, it may not have resulted in a large or problematic increase in loudness and might not affect fitting procedures based on loudness which, in this study, used the CAM2 prescription method.

Preference ratings were consistent with previous literature which found that access to extended bandwidth did not degrade the perceived sound quality for speech stimuli for most participants (Arbogast et al., 2019; Brennan et al., 2014; Füllgrabe et al., 2010; Plyler & Fleck, 2006; Seeto & Searchfield, 2018; Van Eeckhoutte et al., 2020). Results of the present study found that 8 of the 15 participants had some preference for the full bandwidth condition when listening to a running passage of female speech. An additional six participants reported no clear preference for either bandwidth condition. Preference was not correlated with hearing loss or audiometric slope in contrast with some previous studies (Moore et al., 2011; Ricketts et al., 2008). The explanation for this discrepancy is not clear, but speculatively could relate to the audiometric characteristics of the participants differing across studies or differences in tasks. It is also unknown if these preferences for full bandwidth of female speech in quiet would be maintained with other more complex stimuli such speech in noise and babble, or in response to louder sounds like party noise.

Listeners’ preference ratings and their real-world trial settings were within 6.3 dB RMSE from the CAM2 fitting targets for most participants. This may suggest that the direct drive system provided an acceptable fitting using a strategy that aims for broad audible bandwidth. The increase to perceived loudness from the signal produced by the full bandwidth fittings, although audible, did not make a large change to sound levels associated with functional loudness ratings. Coupled with the positive effects on speech recognition and the significantly higher sound quality ratings in the full bandwidth condition (Vaisberg et al., 2021), the battery of outcomes measured in this study points toward the potential value of the provision of full bandwidth fittings as achieved with the direct drive system.

### Study Limitations and Future Directions

A possible limitation to this study is that participants had real-world trials with only the full bandwidth condition without a corresponding narrow bandwidth trial. This may have influenced their preference for the extended bandwidth condition. The goal of this trial was to acclimatize the participants to gain in the extended high frequencies, prior to completing the outcome measures as, on average, their own acoustic hearing aids provided a narrow bandwidth. Future studies could incorporate a design where participants have real-world trials wearing devices in both the narrow bandwidth condition and the full bandwidth condition. In addition, preference ratings were measured with speech stimuli in quiet and may not generalize to other listening situations.

Earlens devices are fitted using vented ear-tips and as such, low-frequency sound presented in the sound field could enter the ear canal through this pathway for at least some of the participants. Measurements of insertion loss are included in supplemental material S3 and indicate that significant vent-transmitted sound was provided for the majority of ears to approximately 800 Hz. Although the goal of this study was to evaluate outcomes with the devices as fitted, future studies could incorporate a design where filtered stimuli are sent to the aids via streaming or direct audio input to determine whether the low-frequency perceptual impacts are provided by the direct drive sound or via the vent, and how this may interact with audiometric configuration. Finally, we note that our outcome measures were completed using the aided conditions only with no unaided comparison. Future studies could include both unaided and aided conditions to demonstrate aided benefit of the direct drive fittings.

## Conclusions

Participants with mild-to-severe sensorineural hearing loss were fitted with the Earlens direct drive hearing devices for a clinical trial period. Broadband fittings were achievable without feedback, and devices were wearable with average requested fine-tuning based on user preference under 3 dB RMSE across frequencies. Compared with a narrowband condition, provision of full bandwidth was beneficial for high-frequency consonant detection and recognition and for sentence recognition in multitalker masking. There was a small but perceivable increase in the loudness with the provision of the full bandwidth. Preference results suggested that the majority of participants either preferred the full bandwidth setting or had no preference between the two bandwidth conditions. These results provide further evidence to support hearing aid fittings which include the provision of extended bandwidth to those with sensorineural hearing loss in the mild-to-severe range.

## Supplemental Material

sj-pdf-1-tia-10.1177_2331216521999139 - Supplemental material for Detection, Speech Recognition, Loudness, and Preference Outcomes With a Direct Drive Hearing Aid: Effects of Bandwidth Click here for additional data file. Supplemental material, sj-pdf-1-tia-10.1177_2331216521999139 for Detection, Speech Recognition, Loudness, and Preference Outcomes With a Direct Drive Hearing Aid: Effects of Bandwidth by Paula Folkeard, Maaike Van Eeckhoutte, Suzanne Levy, Drew Dundas, Parvaneh Abbasalipour, Danielle Glista, Sumit Agrawal and Susan Scollie in Trends in Hearing
