# Supplementary material for: Detection, Speech Recognition, Loudness, and Preference Outcomes With a Direct Drive Hearing Aid: Effects of Bandwidth
Source: Trends Hear. 2021 Apr 19;25:2331216521999139. doi: 10.1177/2331216521999139 (PMC8060758; doi:10.1177/2331216521999139)

S1. Real ear aided response for one participant's personal hearing aid showing output across frequency for a 65 dB SPL speech signal. The maximum audible output frequency (MAOF) range is the highest frequency at which the long-term average (Arrow A) and the peak speech spectrum (Arrow B) is at or above the listener's threshold (Alexander, 2015). This area indicates the fitted bandwidth for this participant's own hearing aid.

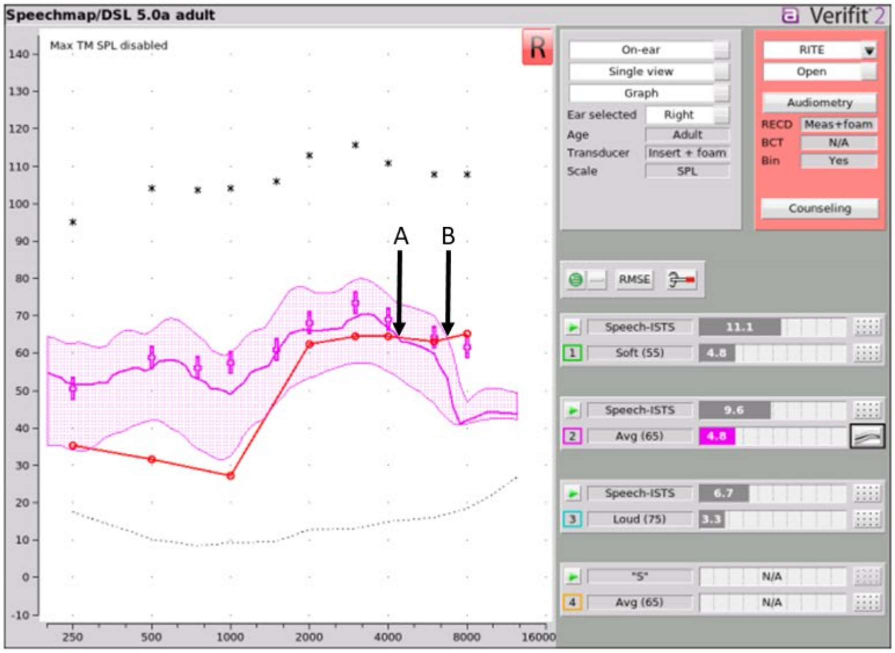

S2. Summary statistics of measured maximum audible output frequencies of the participants' own acoustic hearing aids.

**ISTS speech**	**55 dB SPL**		**65 dB SPL**		**75 dB SPL**	
**MAOF measure**	**RMS**	**Peak**	**RMS**	**Peak**	**RMS**	**Peak**
Mean	2467	4534	4185	5719	5088	6539
Median	2429	4317	4284	5508	4913	6146
Minimum	608	1882	1724	2552	2225	4000
Maximum	4802	10201	9607	10400	9607	10675
SD	1014	1816	1648	1757	1598	1464

S3. Insertion loss of the Earlens devices were measured using the Audioscan VF2. The results are plotted by subtracting the unoccluded response (REUR; no ear-tip in the ear canal) from the

occluded response (REOR; ear-tip in the ear canal and turned off) so that a more negative number indicates greater attenuation of the incoming sound. The mean +/-SD and min/max values are shown for 30 ears (15 participants x 2 ears).

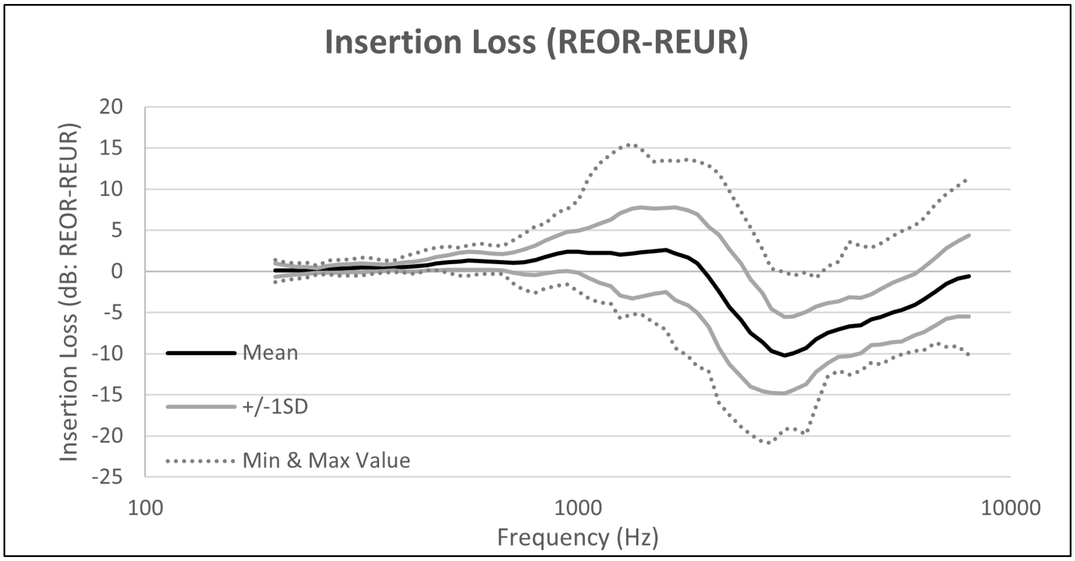

Supplement: sj-pdf-1-tia-10.1177_2331216521999139 - Supplemental material for Detection, Speech Recognition, Loudness, and Preference Outcomes With a Direct Drive Hearing Aid: Effects of Bandwidth [file sj-pdf-1-tia-10.1177_2331216521999139.pdf]
